# Comparing the efficacy in reducing brain injury of different neuroprotective agents following neonatal hypoxia–ischemia in newborn rats: a multi-drug randomized controlled screening trial

**DOI:** 10.1038/s41598-023-36653-9

**Published:** 2023-06-10

**Authors:** Hemmen Sabir, Elke Maes, Margit Zweyer, Yvonne Schleehuber, Farhad B. Imam, Jared Silverman, Yasmine White, Raymand Pang, Anca M. Pasca, Nicola J. Robertson, Emin Maltepe, Maria E. Bernis

**Affiliations:** 1 Deutsche Zentrum für Neurodegenerative Erkrankungen (DZNE) e.v., Venusberg-Campus 1, 53127 Bonn, Germany; 2 Department of Neonatology and Pediatric Intensive Care, Children’s Hospital University of Bonn, Bonn, Germany; 3 Bill & Melinda Gates Foundation, Seattle, WA USA; 4 Gates Medical Research Institute, Boston, MA USA; 5 Department of Pediatrics, The University of California, San Francisco, CA USA; 6 Institute for Women’s Health, University College London, London, WC1E 6HU UK; 7 Division of Neonatology, Department of Pediatrics, Stanford University, Stanford, CA USA; 8 Centre for Clinical Brain Sciences, University of Edinburgh, Edinburgh, EH16 4SB UK

**Keywords:** Neuroscience, Diseases of the nervous system, Stroke

## Abstract

Intrapartum hypoxia–ischemia leading to neonatal encephalopathy (NE) results in significant neonatal mortality and morbidity worldwide, with > 85% of cases occurring in low- and middle-income countries (LMIC). Therapeutic hypothermia (HT) is currently the only available safe and effective treatment of HIE in high-income countries (HIC); however, it has shown limited safety or efficacy in LMIC. Therefore, other therapies are urgently required. We aimed to compare the treatment effects of putative neuroprotective drug candidates following neonatal hypoxic-ischemic (HI) brain injury in an established P7 rat Vannucci model. We conducted the first multi-drug randomized controlled preclinical screening trial, investigating 25 potential therapeutic agents using a standardized experimental setting in which P7 rat pups were exposed to unilateral HI brain injury. The brains were analysed for unilateral hemispheric brain area loss after 7 days survival. Twenty animal experiments were performed. Eight of the 25 therapeutic agents significantly reduced brain area loss with the strongest treatment effect for Caffeine, Sonic Hedgehog Agonist (SAG) and Allopurinol, followed by Melatonin, Clemastine, ß-Hydroxybutyrate, Omegaven, and Iodide. The probability of efficacy was superior to that of HT for Caffeine, SAG, Allopurinol, Melatonin, Clemastine, ß-hydroxybutyrate, and Omegaven. We provide the results of the first systematic preclinical screening of potential neuroprotective treatments and present alternative single therapies that may be promising treatment options for HT in LMIC.

## Introduction

Neonatal encephalopathy (NE) remains one of the leading causes for child mortality and is a major contributor to long term morbidities, including epilepsy, cerebral palsy and neurocognitive delays^1,2^. In industrialized countries the incidence of NE is 0.5–1.5 per 1000 live births^3^; here therapeutic hypothermia (HT) is standard treatment, reducing mortality and long-term morbidities by 20–25%, with a number needed to treat of seven^2^. However, the burden of NE secondary to intrapartum events is disproportionately higher amongst low- and middle-income countries (LMIC). In particular, sub-Saharan Africa and Southeast Asia account for more than 85% of all NE cases worldwide with regional estimates as high as 14.9 per 1000 live births^4^. Potential therapies are limited in LMIC as the feasibility, safety and efficacy of HT in all LMIC remains uncertain^5^. These differences in response to HT may originate from the timing of the HI insult (longer intrapartum insults with early presentation of seizures within 2 h of birth), comorbidities such as poor nutrition and the co-existence of infection/inflammation as seen in studies in sub-Sahara Africa^6^. Therefore, identifying alternative neuroprotective treatments to HT is of major global interest, as it will have a large impact on the global burden caused by NE.

The pathophysiology following neonatal hypoxic-ischemic brain injury is complex and evolves through several different phases. A reduction in placental blood flow and fetal oxygen supply occurs in the first phase^7^; such vasculature chances lead to a loss of cerebral autoregulation with a reduction of systemic fetal blood pressure^7^. The decrease of oxygen and depletion of energy causes increased excitotoxicity, an increase of intracellular calcium, oxidative stress and mitochondrial dysfunction^7^, leading to primary energy failure and some apoptotic and necrotic cell death. After reperfusion at resuscitation, the initial hypoxia-induced cytotoxic oedema and accumulation of excitatory amino acids partially resolves, with apparent recovery of cerebral oxidative metabolism. It is thought that the neurotoxic cascade is largely inhibited during the latent phase. Around 6–24 h after HI, a secondary phase occurs despite adequate oxygenation and perfusion. This secondary phase is associated with failure of cerebral oxidative metabolism (secondary energy failure), further glutamate driven excitotoxicity, inflammation, free radical and reactive oxygen species (ROS) production and mitochondrial dysfunction^8^. The third phase, lasting days to weeks or months, is characterized by chronic inflammation, which may lead to epigenetic changes and impairs neurogenesis, synaptogenesis and axonal growth^9^.

Many of the putative therapies for NE target specific pathological injury processes, including excitotoxicity, oxidative stress, overproduction of reactive oxygen species (ROS), mitochondrial dysfunction, neuroinflammation and regeneration^10^. HT-induced cellular protection, however, is a global process affecting multiple molecular and cellular mechanisms and results in a 6–10% decrease in cerebral metabolism for every 1 °C reduction in body temperature^11^. However, as there are concerns around the safety and efficacy of HT in LMIC, we focus on economical, simple single therapies as an alternative to HT in this study.

There are currently no other safe and effective therapeutic options for NE apart from HT. Some agents are currently under evaluation in randomized-controlled trials (RCTs) in combination with or without HT, for example erythropoietin (clinical trials gov: NCT02811263), allopurinol (clinical trials gov: NCT03162653), topiramate (clinical trials gov: NCT01765218), caffeine (clinical trials gov: NCT03913221), melatonin (clinical trials gov: NCT03806816) or stem cells (clinical trials gov: NCT02612155). Some very potent pre-clinical agents such as inhaled xenon and erythropoietin have failed to show significant neuroprotection in clinical trials when combined with HT^12,13^. One of the challenges transferring in-vivo or in-vitro studies into clinical studies is that preclinical studies are often performed in different labs on different continents, using different treatment protocols and models. In addition, they often lack comparison to RCTs regarding experimental design and statistical analysis^14^. The Vannucci rodent model of unilateral brain injury is an established animal model used by researchers worldwide to screen for neuroprotective potency of agents, understand the underlying pathophysiological mechanisms and study the different mode of actions following neonatal hypoxic-ischemic brain injury^15^. However, there is considerable variability in the model with groups using different concentrations and duration of hypoxia^15^. Often, pertinent experimental details such as temperature control during treatment or hypoxia are not reported and the number of animals used not reported^16^. This challenges researchers to reproduce results although using the “same” animal model and questions the validity of the published results.

As previously stated, different treatment options have shown neuroprotection using the Vannucci model. In addition, the Vannucci model has set the basis for hypothermic neuroprotection, which has become standard care. Nevertheless, this model has never been used to prove efficacy and compare the neuroprotective potency of multiple different potential neuroprotective agents in a standardized single lab set-up.

The aims of this study were: (i) screen the literature for preclinical and clinical studies of promising putative therapeutic agents for NE; (ii) establish a rank-order for the most promising neuroprotective treatments which could be used in a LMIC setting by using specific scores based on evidence for an efficiency treatment; (iii) Test and directly compare neuroprotective efficacy of 25 therapeutic agents using the standardized Vannucci P7 rat model in an established single lab set-up to identify the most promising agents to progress to more intensive studies in large animal models.

## Materials and methods

### Literature search strategy and prioritization of drug candidates

In September 2019 a comprehensive literature search was performed using PubMed database, Google Scholar and Clinivaltrials.gov. The literature search incorporated all studies utilizing potentially applicable drug candidates for LMICs and included the terms “newborn”, “hypoxia–ischemia”, “brain injury”, “neuroprotection”, “neonatal encephalopathy”. Based on this first search, an initial list of 33 neuroprotective drug candidates was created. The following information for each drug candidate was provided within the list: mode of action, dosing schedule, route and timing of administration, model description (in vitro model, in vivo model, and details on clinical studies), population description, inclusion criteria, outcome measures, detection of efficacy, detection of adverse events, overall findings and conclusions. In addition, each drug candidate was assessed for the following delivery-related information: ability to cross the placenta, ability to cross the blood brain barrier, demonstrated routes of administration and cold chain requirements, as well as availability and cost (Supplementary Table 1). Available pharmacokinetics and safety data were also identified for each candidate. From this list of compounds identified in the literature, priority review was assigned to compounds with neuroprotective efficacy in at least two different laboratories and/or models. Each of these compounds was subsequently assigned for evaluation by at least 2 independent scientific reviewers which assigned a score of 1–9, where 1 is considered as enough evidence for an efficiency treatment and 9 not enough evidence for efficiency treatment. The literature search was repeated in March 2022 and two additional drug candidates were added based on a broader definition of neuroprotective activity (Clemastine and Sonic Hedgehog Agonist), increasing the total number of candidates to 35 (Supplementary Table 1).

### Scoring and ranking of drug candidates

In December 2019 and March 2022 a round table meeting of the authors, plus additional consulted experts, was held to achieve consensus on scoring and ranking of drug candidates, in addition to review of the treatment protocols to be used in this platform. Each available candidate from the initial literature search was scored based on strength of efficacy evidence, magnitude of treatment effect, strength of safety evidence, knowledge of pharmacokinetics and practicality for implementation in LMIC based on route of administration, timing constraints with respect to injury, cold chain requirement and anticipated cost (Supplementary Table 1). Not all compounds from this final list were assessed as being tested in the model (e.g. xenon, deprioritized due to limitations for scaling in LMIC health systems).

Based on the scoring of each candidate, a prioritized list was created for in vivo testing within-laboratory comparison on the platform described based on a scoring from 1 to 9 as mentioned above (Supplementary Table 1). Due to feasibility and laboratory capacity, a total of 25 putative therapeutic agents were tested and results compared within an initial screening phase using the established Vannucci P7 rat model in a single lab. Progress toward complete evaluation of the candidate list was significantly delayed during the COVID-19 pandemic due to limitations on laboratory activity and staffing.

### Protocol for individual drug candidates

The initial dose for each candidate drug was determined based on best available evidence from the literature and previous usage in the Vannucci P7 rat model (preferred) or other rodent models (Table 1, Supplementary Table 3, including references). In order to maximize the potential to identify active agents in this first screening phase, in which drug candidates could be potentially administered before the onset of hypoxia–ischemia (antenatally), it was decided that the first dosage of drug administration was given before hypoxia for drugs known to cross the placenta (Table 1, Supplementary Table 3 including references). For all other therapeutic agents, the first administration was after hypoxia. All drug candidates were administered intraperitoneally (i.p., 0.1 ml/10 g body weight) and dosing interval was based on putative mechanism of action and pharmacokinetic parameters. Dosing regimens were designed to cover the first 72 h after birth, the time window used to cool newborns with NE (Table 1).

**Table 1:** List of drug candidates.

	Drug dose concentration per injection	Drug dose treatment
2-Iminobiotin	50 mg/kg	1 h before hypoxia, 12, 24, 36, and 48 h after 1st dose
Allopurinol	100 mg/kg	1 h before hypoxia, 12, 24, 36, and 48 h after 1st dose
Azithromycin	45 mg/kg 1st dose, then 22.5 mg/kg	1 h before hypoxia, 24, and 48 h after 1st dose
Caffeine	40 mg/kg	1 h before hypoxia, 24, and 48 h after 1st dose
Carnitine	100 mg/kg	1 h before hypoxia, 24, and 48 h after 1st dose
Cannabidiol	1 mg/kg	1 h before hypoxia, 24, and 48 h after 1st dose
Clemastine	10 mg/kg	Immediately after hypoxia–ischemia, 24, 48, 72, 96, 120 and 144 h after 1st dose
Darbepoietin delayed treatment	25 µg/kg	24 h after hypoxia–ischemia
Darbepoietin immediate treatment	25 µg/kg	1 h after hypoxia–ischemia
Edaravone	9 mg/kg	1 h before hypoxia, 24, and 48 h after 1st dose
Erythropoietin delayed treatment	5000 IE/kg	24, 48, and 72 h after hypoxia–ischemia
Erythropoietin immediate treatment	5000 IE/kg	1 h after hypoxia–ischemia, 24, and 48 h after 1st dose
Iodide	3 mg/kg	1 h before hypoxia, 24, and 48 h after 1st dose
Levetirazetam	20 mg/kg	1 h before hypoxia, 24, and 48 h after 1st dose
Melatonin	25 mg/kg	Immediately after hypoxia–ischemia, 12, 24, 36 and 48 h after 1st dose
Metformin	20 mg/kg	1 h before hypoxia, 24, and 48 h after 1st dose
Magnesiumsulfate	500 mg/kg	1 h before hypoxia, 24, and 48 h after 1st dose
Mitoquinol	0.4 mg/kg	1 h before hypoxia, 12, 24, 36, and 48 h after 1st dose
*N*-Acetylcystein	200 mg/kg	1 h before hypoxia, 12, 24, 36, and 48 h after 1st dose
Omegaven	750 mg/kg	Immediately after hypoxia–ischemia
Omegaven 2 doses	750 mg/kg	Immediately after hypoxia–ischemia and 1 h after 1st dose
Phenobarbital	20 mg/kg	1 h before hypoxia, 24, and 48 h after 1st dose
Sonic Hedgehog Agonist	50 mg/kg	Immediately after hypoxia–ischemia
Sildenafil	10 mg/kg	1 h before hypoxia, 24, and 48 h after 1st dose
ß-Hydroxybutyrate	630 mg/kg	Immediately after hypoxia–ischemia, 2, 5 and 12 h after 1st dose
Tetrahydrobiopterin	20 mg/kg	Immediately after hypoxia–ischemia, 24 and 48 h after 1st dose
Therapeutic hypothermia	–	Immediately after hypoxia–ischemia, body temperature 32 °C for 5 h
Topiramate	40 mg/kg	Immediately after hypoxia–ischemia, 12, 24, 36 and 48 h after 1st dose
Uridine	500 mg/kg	1 h before hypoxia, 24 and 48 h after 1st dose

The individual dosages and drug treatment intervals were based on the current literature (Supplementary Table 3).

Drug candidates were all administered intraperitoneally at different time points covering the perinatal phase.

### Vannucci rat model of unilateral hypoxic–ischemic brain injury

All animal experiment protocols were approved by the animal protection committee from the State Environment Agency of North Rhine-Westphalia, Germany (LANUV) (Approval No. AZ 81-02.04.2018.A166), and all experiments were performed following relevant guidelines and regulations and in compliance with the ARRIVE guidelines. We used our established rat model of unilateral hypoxic-ischemic brain injury, in which we have previously shown significant neuroprotection using HT and additional treatments with immediate and delayed treatment onset following HI^17–21^.

In brief, seven-day old (P7) Wistar rat pups of both genders were used in all our experiments. No animals were excluded prior to the experiments and all pups were born and kept at the central animal laboratory of the Deutsches Zentrum für Neurodegenerative Erkrankungen (DZNE) Bonn, Germany, with a 12:12 h dark/light cycle at an environmental temperature of 21 °C with food and water ad libitum. Litter sizes were between 8 and 15 pups/litter. As previously described, all animals for each experiment were randomized across litter, sex, and weight before the experiments commenced and all read-out analysis were performed by observers blinded to the different treatments. All animals underwent unilateral ligation of the left common carotid artery under general anaesthesia as previously described in detail in our previous publications^17–21^. Animals were kept at physiological temperature whilst handling and ligation, as previously described^17–21^. After ligation of all pups, pups were exposed to 8% oxygen (hypoxia) for 90 min at a rectal temperature (T_rectal_) of 36.0 °C in a temperature-controlled chamber, resulting in a unilateral moderate hypoxic-ischemic insult. Following hypoxia, all pups were kept at T_rectal_ of 37 °C for 5 h at 21% oxygen (except the HT group, which was kept at T_rectal_ of 32 °C for 5 h at 21% oxygen), representing the normothermia group in all our previous studies, before all pups were returned to their individual dams^17–21^. As previously described, temperature during hypoxia and normothermia treatment was continuously measured in “sentinel” pups carrying a rectal temperature probe (IT-21, Physitemp Instruments, Clifton, NJ) or a skin probe (CritiCool,MTRE) on the abdomen. T_rectal_ of 37.0 °C ± 0.2 °C was maintained with a servo-controlled mat (CritiCool, MTRE)^21^. All animals were checked for weight gain and well-being daily.

Each individual experiment included one HI group which received i.p. saline injections (HI/saline) and experimental groups which received the drug candidates i.p. as per protocol (HI/drug candidate—Supplementary Table 2). Each group consisted of 7–14 animals, previously shown to be an adequate group size using the Vannucci model^15^.

### Histopathology and area measurement

After seven days of survival, transcardiac perfusion with phosphate-buffered saline (PBS), followed by 4% paraformaldehyde (Sigma-Aldrich) was performed and the brains were postfixed in 4% paraformaldehyde overnight at 4 °C and embedded in paraffin. Embedded brains were coronally cut in 10 μm sections followed by staining with haematoxylin and eosin (H&E). As previously described, global brain area tissue loss was calculated by measurement of brain areas in ipsilateral and contralateral hemispheres of two sections, from two neighbouring blocks (− 3.72 ± 0.7 mm from Bregma) using ImageJ software (ImageJ, version 1.46r, National Institutes of Health, Bethesda, MD, United States)^17^. Tissue loss was analysed by two individuals blinded to the treatments.

As previously described, brain area loss correlates very strongly with histopathological analysis^17^. Moderate brain injury was defined as a median brain area loss within the treatment group between 35 and 50% in the HI/saline group^17^. For quality standard reasons and comparability of results, experiments with a median brain area loss of < 35% or > 50% in the HI/saline group were excluded (n = 2).

### Statistical modelling

The model used in this study incorporated independent experiments, in which active compounds were tested against a common saline comparator. To facilitate borrowing of non-concurrent control data into the analysis of the individual treatments, while considering the study-to-study variability, we employed a linear mixed effect model with a fixed treatment effect and an experiment random effect^22^. Specifically, if ${y}_{ij}$ denotes the brain area loss percentage of the *j*th rat in the *i*th experiment, we model

$${y}_{ij}|{\alpha }_{i}= \mu + {\alpha }_{i}+\sum \limits_{k}{\beta }_{k}1\left\{{\mathrm{Trt}}_{i}=k\right\}+ {\mathcal{E}}_{ij},$$

where $\mu$ is the mean control area loss percentage, ${\alpha }_{i}\sim \mathcal{N}(0,{\tau }^{2})$ is the experiment random effect, ${\beta }_{k}$ is the *k*th treatment effect, ${\mathrm{Trt}}_{i}$ denotes the active compound given in the *i*th experiment, and ${\mathcal{E}}_{ij}\sim \mathcal{N}(0,{\sigma }^{2})$ is a random error.

Further, we used a Bayesian implementation of the above model from the *brms* library in the 4.1.2 version of the R statistical software^23^. The default implementation of this library uses improper flat prior distributions for model parameters, and we saw no need to change it. Markov Chain Monte Carlo (MCMC) samples from the posterior distribution are then generated using Stan. The respective treatment effects were then summarized in the form of posterior medians and 95% Bayesian credible intervals, using the sample 2.5%, 50% and 97.5% percentiles. In addition, posterior efficacy of each treatment $k=1,\dots ,K$ was evaluated using the Monte Carlo approximation

$${\mathbb{P}}\left({\beta }_{k}<0|\mathrm{data}\right)\approx \frac{1}{N}\sum \limits_{i=1}^{N}1\left\{{\beta }_{k}^{\left(i\right)}<0\right\},$$

where ${\left\{{\beta }_{\left\{k\right\}}^{\left(i\right)}\right\}}_{\left\{i=1\right\}}^{N}$ denotes the size $N$ posterior sample of the *k*th treatment effect. Our reported estimates were based on four independent MCMC samples of size 10,000 each, of which the first 5000 were discarded as “burn in” period.

### Exclusion of outlier experiments

To determine whether one or more litters should be excluded from the analysis due to abnormally small or large observed values, we took the following approach:

1. Conduct Kruskal–Wallis test on control rats to determine whether there is a difference in the distribution of brain area loss across litters. If the null hypothesis of no differences cannot be rejected at the 5% level, the process stops. Otherwise continue to the next step.
2. For $j={argmax}_{1\le i\le K}\left\{{\overline{R} }_{i}-\frac{n+1}{2}\right\}$, where ${\overline{R} }_{i}$ denotes the average rank among rats from the *i*th litter (when the full sample is sorted in an increasing order) $K$ is the number of different litters and $n$ the total number of control rats, calculate the contrast
  $${c}_{i}=\left\{\begin{array}{ll}\frac{1}{K-1}& i\ne j\\ & \\ -1& i=j\end{array}\right.,$$
  for $1\le i\le K$, and the subsequent test statistic
  1 $$T={\left(\frac{\sum \limits_{i=1}^{K}{{\varvec{c}}}_{{\varvec{i}}}{\overline{R} }_{i}}{SE}\right)}^{2},$$
  where $\mathrm{SE}$ is the approximate standard error
  $$\mathrm{SE}=\sqrt{\frac{n(n+1)}{12}{\sum }_{i=1}^{K}\frac{{{\varvec{c}}}_{{\varvec{i}}}^{2}}{{n}_{i}}},$$
  where ${n}_{i}$ is the size of the *i*th litter. Note that (1) is a measure of the distance of the most deviant litter from the rest.
3. Perform a permutation test by randomly shuffling the existing observations between the different litters for a large number ($N$) of permutations and recording the same test statistic (1) as in Step 2 each time. Denote by ${T}_{i}$ the recorded test statistic for the *i*th permutation, $1\le i\le N$. The p-value of this test is then approximately
  2 $$\frac{1}{N}\sum \limits_{i=1}^{N}1\left\{{T}_{i}>T\right\}.$$
  If this approximate p-value (1) exceeds 0.05 stop, otherwise exclude the *j*th litter from all future analyses and return to Step 1.

The process then repeats itself until at one point it is forced to stop.

### Use of experimental animals

Animal experiments were conducted in accordance with the animal protection committee from the State Environment Agency of North Rhine-Westphalia, Germany (LANUV, AZ 81-02.04. 2018.A166) and following the ARRIVE guidelines.

## Results

### Scoring and ranking of drug candidates

The overall ranking list consisted of 35 drug candidates (Supplementary Table 1). Based on the analysis/scoring by two independent researchers (see “Materials and methods” section), 25 drug candidates were chosen, as this was the maximum capacity of candidates to be used in the single-lab set-up. The 25 drug candidates that were chosen to be evaluated and tested for neuroprotection in the Vannucci model are listed in Table 1 and Supplementary Table 3. HT was included as the “gold standard” neuroprotective treatment in our P7 rat model.

### Results from the Vannucci rat model

A total of 20 experiments consisting of 45 experimental groups (n = 20 HI/Saline) were performed between April 2020 and July 2022 and a total of 606 animals used (n = 304 male, 302 female). No animals were excluded prior to the experiments. The mortality rate was 62/584 during the experimental phase (related to surgical complications (n = 35) or death during hypoxia (n = 27)). The remaining animals were either randomized to the HI/saline group (pooled n = 230) or the HI/drug compound groups (see Table 1 for dosage and frequency of treatment). Each treatment group consisted of 7–14 P7 rat pups, as per expected variability within this animal model. As presented in Table 2, median pooled brain area loss in the HI/saline group was 41.46% (range 0.00–64.97%, n = 230), representing a moderate brain injury in our rat model. Table 2 presents the results of median brain area loss for each tested drug candidate versus the HI/saline group in the individually performed experiments (matched placebo). We found that Sonic Hedgehog Agonist, Caffeine, Melatonin, Allopurinol, Clemastine and Omegaven significantly reduced median brain area loss in the individual experimental setup. Additionally, therapeutic hypothermia also significantly reduced median brain area loss compared to matched placebo.

**Table 2:** Median brain area loss.

	Median brain area loss HI control (range)	Median brain area loss compound (range)	p-value
Sonic Hedgehog Agonist	35.42 (4.47–36.89; n = 12)	5.74 (0.36–34.14; n = 14)	**0.0011**
Caffeine	44.04 (1.65–50.29; n = 12)	7.31 (0.00–18.35; n = 12)	**0.0014**
Melatonin	46.79 (32.02–63.41; n = 13)	32.93 (1.29–55.44; n = 15)	**0.0061**
Allopurinol	39.01 (0.00–60.54; n = 13)	17.73 (0.00–34.61; n = 9)	**0.0207**
Clemastine	49.31 (0.41–64.97, n = 11)	25.00 (1.75–52.57, n = 11)	**0.0209**
Therapeutic hypothermia	42.19 (24.24–60.69; n = 10)	28.96 (14.96–43.29; n = 11)	**0.0295**
Omegaven	39.80 (25.50–48.76; n = 10)	28.94 (3.32–48.75; n = 12)	**0.0405**
Omegaven 2 doses	39.80 (25.50–48.76; n = 10)	32.03 (6.73–47.24; n = 11)	0.0607
Tetrahydrobiopterin	44.75 (5.01–58.22; n = 11)	35.19 (0.00–54.11; n = 10)	0.1210
Erythropoietin delayed treatment	36.84 (24.45–47.74; n = 11)	34.09 (7.25–39.24; n = 7)	0.1385
*N*-Acetylcystein	48.26 (40.10–57.40; n = 11)	37.21 (3.35–59.93; n = 13)	0.1500
ß-Hydroxybutyrate	38.69. (0.00–54.40; n = 10)	28.22. (0.00–55.13; n = 12)	0.2337
Phenobarbital	37.37 (0.00–58.30; n = 12)	46.72 (0.00–55.97; n = 9)	0.2765
Magnesiumsulfate	49.11 (30.14–56.21; n = 8)	44.01 (10.31–51.14; n = 10)	0.3476
Levetirazetam	37.37 (0.00–58.30; n = 12)	40.88 (17.27–55.43; n = 10)	0.3715
Erythropoietin immediate treatment	36.84 (24.45–47.74; n = 11)	35.65 (15.67–49.48; n = 8)	0.4366
Carnitine	40.44 (4.63–57.48; n = 12)	45.92 (2.78–59.68; n = 14)	0.4624
2-Iminobiotin	44.75 (5.01–58.22; n = 11)	50.92 (10.31–71.14; n = 11)	0.4996
Darbepoietin immediate treatment	41.25 (9.46–60.06; n = 11)	45.16 (16.85–55.30; n = 13)	0.5309
Edaravone	37.37 (0.00–58.30; n = 12)	39.17 (2..62–58.03; n = 11)	0.5541
Mitoquinol	38.69. (0.00–54.40; n = 10)	25.83. (0.05–55.71; n = 11)	0.5573
Cannabidiol	49.11 (30.14–56.21; n = 8)	47.58 (2.01–58.48; n = 9)	0.5877
Iodide	31.76 (27.24–54.31; n = 12)	32.69 (0.76–47.95; n = 14)	0.5952
Darbepoietin delayed treatment	41.25 (9.46–60.06; n = 11)	44.36 (2.91–66.72; n = 14)	0.6475
Metformin	44.34 (7.25–63.92; n = 14)	48.93 (13.51–66.54; n = 13)	0.7564
Sildenafil	44.84 (30.32–62.89; n = 14)	43.83 (1.39–57.40; n = 12)	0.8201
Topiramate	38.69. (0.00–54.40; n = 10)	36.83 (0.01–60.11; n = 11)	0.9725
Azithromycin	39.15 (22.73–60.24; n = 13)	43.96 (7.50–58.99; n = 14)	0.9810
Uridine	35.78 (2.58–50.99; n = 10)	43.15 (34.23–55.82; n = 11)	0.0197
Pooled	**41.46 (0.00–63.92, n = 230)**		

Individual median brain area loss results for each tested drug candidate.

Pooled median brain area loss in the HI/saline group was 41.46% (range 0.00–64.97; n = 230).

Significant values are in bold.

**Figure 1:**
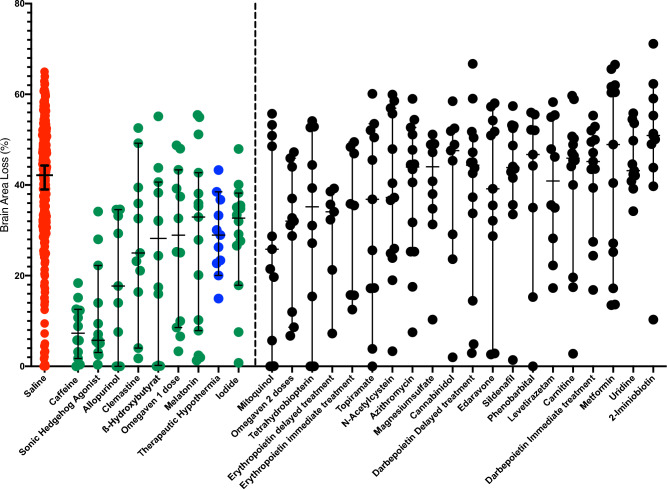
Individual box plots of percentage of brain area loss for experimental treatments and the pooled HI/saline group. In red is the data for all HI/Saline treated animals while green indicates the eight drugs which significant reduced brain area loss. Blue corresponds to the standard neuroprotective treatment (therapeutic hypothermia).

Figure 1 presents the individual box plots of percentage of brain area loss for experimental treatments and the pooled HI/saline group.

For final analysis, as described in the methods, a statistical modelling approach was used including all treatment groups to test for efficacy of reduction of brain area loss against the pooled HI/saline group. We found that eight compounds significantly reduced brain area loss with the strongest treatment effect for Caffeine, sonic hedgehog agonist (SAG) and Allopurinol (probability of efficacy of 100% compared either to the individual HI/saline control or pooled HI/saline control groups), followed by Melatonin (probability of efficacy of 99.90% compared either to the individual HI/saline control or pooled HI/saline control groups) and Clemastine (probability of efficacy of 99.70% compared either to the individual HI/saline control or pooled HI/saline control groups). We also identified compounds that were active compared to the more robust pooled placebo data that would have been missed on the basis of individual experiments: ß-Hydroxybutyrate (probability of efficacy of 99.50% compared to the pooled HI/saline control groups; non-significant probability of efficacy of 86% when compared to the same experiment Hi/saline group), Omegaven (probability of efficacy of 98.90% compared to the pooled HI/saline control groups; non-significant probability of efficacy of 96.8% when compared to the same experiment Hi/saline group), and Iodide (probability of efficacy of 98.0% compared to the pooled HI/saline control groups; non-significant probability of efficacy of 93.3% when compared to the same experiment Hi/saline group) (Table 3). Serving as an internal neuroprotective control, therapeutic hypothermia also significantly reduced brain area loss (probability of efficacy of 98.80% compared either to the individual HI/saline control or the pooled HI/saline control groups). Of major interest, probability of efficacy was superior to therapeutic hypothermia for Caffeine, SAG, Allopurinol, Melatonin, Clemastine, ß-Hydroxybutyrate, and Omegaven. None of the other tested candidates demonstrated statistically significant efficacy (Table 3).

**Table 3:** Probability of efficacy for all 25 tested candidates.

Treatment	Vs. Concurrent control		Vs. Pooled HI/saline	
	Effect estimate (95% CrI) individual control group	Pr (efficacy) (%)	Effect estimate (95% CrI) pooled HI/saline group	Pr (efficacy) (%)
Caffeine	− 21.5 (− 28.5; − 14.6)	100.00	− 22.3 (− 31.9; − 12.7)	**100.00**
Sonic Hedgehog Agonist	− 18.9 (− 27.9; − 9.8)	100.00	− 25.7 (− 34.8; − 16.1)	**100.00**
Allopurinol	− 18.3 (− 35.1; − 1.4)	98.30	− 19.5 (− 30; − 9)	**100.00**
Melatonin	− 19.2 (− 31.2; − 7)	99.80	− 13.8 (− 23.2; − 4.8)	**99.90**
Clemastine	− 18.4 (− 33.6; − 2.8)	99.80	− 13.9 (− 24.3; − 3.9)	**99.70**
ß-Hydroxybutyrate	− 9.1 (− 26.4; 7.7)	86.00	− 12.9 (− 22.2; − 3.2)	**99.50**
Omegaven 1 dose	− 9.3 (− 19.1; 0.5)	96.80	− 10.8 (− 19.9; − 1.7)	**98.90**
Therapeutic hypothermia	− 14 (− 23.9; − 4.3)	99.50	− 11.1 (− 21; − 1.5)	**98.80**
Iodide	− 7.1 (− 16.6; 2.5)	93.30	− 9.2 (− 17.9; − 0.4)	**98.00**
Mitoquinol	− 6 (− 25.6; 13.1)	73.60	− 9.7 (− 19.6; 0.4)	97.10
Omegaven 2 doses	− 7 (− 16.5; 2.3)	93.00	− 8.5 (− 17.9; 0.9)	96.20
Tetrahydrobiopterin	− 9.3 (− 23.5; 4.6)	90.40	− 7.4 (− 17.3; 2.3)	93.10
Erythropoietin delayed treatment	− 7.1 (− 16.5; 2.2)	93.50	− 8.7 (− 20.2; 2.9)	92.90
Erythropoietin immediate treatment	− 4.3 (− 15.6; 6.9)	78.00	− 6 (− 16.8; 5.1)	85.80
Topiramate	− 1.4 (− 20.1; 17.2)	55.70	− 5 (− 14.8; 5)	83.60
*N*-Acetylcystein	− 10.8 (− 22.3; 0.9)	96.70	− 3.6 (− 13.5; 5.7)	76.00
Azithromycin	− 1.9 (− 13; 8.9)	63.70	− 0.5 (− 9.2; 8.1)	54.10
Magnesiumsulfate	− 5.5 (− 17.1; 5.9)	83.80	− 0.4 (− 11.1; 9.9)	52.70
Cannabidiol	− 5.8 (− 20.4; 8.7)	79.30	− 0.4 (− 11.4; 10.4)	52.60
Darbepoietin delayed treatment	0.6 (− 13.6; 14.9)	46.50	0.1 (− 8.8; 9)	49.20
Edaravone	5.2 (− 12.1; 22)	26.60	0.3 (− 9.6; 10.7)	48.20
Sildenafil	− 1.6 (− 11.6; 8.2)	62.80	1.8 (− 7.7; 11.1)	34.80
Phenobarbital	7.4 (− 10.9; 25.7)	20.50	2.4 (− 8.2; 13.4)	33.50
Levetirazetam	7.9 (− 7.9; 23.8)	15.60	2.9 (− 7.2; 13.5)	28.40
Carnitine	3.9 (− 9.1; 17)	27.00	2.6 (− − 6.2; 11.6)	28.00
Darbepoietin immediate treatment	3.8 (− 7.8; 15.6)	25.50	3.3 (− 5.7; 12.3)	23.20
Metformin	1.9 (− 14; 17.9)	40.4	3.6 (− 5.8; 12.9)	21.7
Uridine	8.9 (1.3; 16.4)	1.00	7.4 (− 2; 16.9)	6.20
2-Iminobiotin	4.2 (− 9.4; 17.5)	26.10	8.8 (− 1.6; 18.6)	4.80

We found that eight drug components significantly reduced brain area loss compared to the pooled HI/saline group with the strongest treatment effect for Caffeine, sonic hedgehog agonist and Allopurinol, followed by Melatonin, Clemastine, ß-Hydroxybutyrate, Omegaven, and Iodide. Probability of efficacy was superior to therapeutic hypothermia for Caffeine, Sonic Hedgehog Agonist, Allopurinol, Melatonin, Clemastine, ß-Hydroxybutyrate, and Omegaven. Left two columns present effect estimates compared to the individual experimental control group. Right two columns present effect estimates compared to the pooled HI/saline group.

Significant values are in bold.

## Discussion

This study presents the first multi-drug neonatal preclinical randomized control trial (RCT) in P7 rats comparing efficacy of putative therapeutic agents for brain protection after HI. Our goal was to perform a head-to-head screen of compounds previously reported to have neuroprotective activity to identify candidates for more intensive future study (e.g. mechanism of action, pharmacokinetic/-dynamic relationships, and large animal models). Using the established and standardized Vannucci model, we identified eight therapies providing significant neuroprotection, based on significant reduction of global brain tissue loss after 7 days survival. We compared treatment efficacy against HT, the current “gold standard” therapy in our model, which has not been yet performed systematically in any other study. We found strongest evidence for significant neuroprotection for the following compounds: Caffeine, SAG, Allopurinol, Melatonin, Clemastine and Omegaven. ß-Hydroxybutyrate and Iodide were found to be significantly neuroprotective only comparing against the pooled HI/saline group, but failed to be significantly neuroprotective against the matched control group.

The unique aim of this study was to use a standardized pre-clinical animal model with proven hypothermic neuroprotection and to test the currently available potential neuroprotective treatment compounds described in the literature in a standardized experimental set-up. Therefore, a list of 25 treatment candidates was selected based on a scoring system, including pre-clinical and clinical evidence, best representing the currently available neuroprotective treatment compounds following neonatal hypoxic-ischemic brain injury. Eight agents displayed notable activity in our screen.

### Caffeine

Caffeine, a non-specific adenosine-receptor antagonist, is widely used in neonates to treat apnoea^24^. The neuroprotective potency of caffeine has already been suggested in a clinical randomized controlled study treating apnoea of prematurity^25^. In the secondary analysis of the study, newborns from the caffeine treatment group showed reduced cerebral palsy and cognitive delay. In the context of neonatal HI, caffeine has shown neuroprotection in small and large preclinical animal models^26^. Caffeine has proven long term neuroprotection when administered before and after neonatal HI in preclinical models in different dosages^26^. We found that 40 mg/kg administered intraperitoneal in three doses (Table 1) (before HI, 24 h and 48 h after HI) significantly reduced global brain tissue loss. Compared to the literature and clinical standard dosing regimens in neonates, our dosing was higher. However, studies in preterm infants have shown that higher doses of caffeine are effective without reporting significant side effects when compared to the standard treatment^27–29^. In our study, we did not note any adverse effects, like increased seizure rates, weight loss or death, in the animals treated with caffeine when we used a dose of 40 mg/kg/dose. Of note, we administered caffeine intraperitoneal and not intravenous, as per standard in neonates. The primary mode of action for caffeine includes reduction of excitotoxicity, reduction of inflammation and reduction of astrogliosis, all resulting in improved long term neurocognitive and motor outcome in pre-clinical studies^30–32^. In our multi-drug study caffeine showed superior neuroprotection compared to all other compounds used in this study. Especially when comparing the neuroprotective potency against therapeutic hypothermia, caffeine significantly demonstrated a further reduction in brain area loss. Caffeine treated animals showed a significantly reduced variability of brain injury. In general, the variability of brain injury in this model is known to be high^15^, but caffeine treatment was able to reduce the variability even though the wide variability was still observed in the matched control group.

### Sonic Hedgehog Agonist (SAG)

SAG is an agonist of the Sonic Hedgehog signalling pathway which regulates many processes of development during central nervous system (CNS) maturation. Specifically, it has been shown to be crucial for neuronal circuit and synapse formation^33^, brain vascularisation^33^ and oligodendrogenesis^34^. Recently, it has also been shown that SAG treatment prevents cerebellar injury and is neuroprotective using a neonatal stroke model without interfering with CNS development^35^. Therefore, although SAG did not meet all of our initial selection criteria, it was tested in the HIE model. Of importance, a single dose treatment was used in a neonatal stroke model and showed significant neuroprotection with long-term beneficial effects in newborn rats^35^. We confirm in this current study, that a single dose of 50 mg/kg (Table 1) after hypoxia is sufficient for significant neuroprotection in our standardized model following neonatal hypoxic-ischemic brain injury.

### Allopurinol

Allopurinol, a xanthine oxidase inhibitor and free radical scavenger, has been shown to be neuroprotective following neonatal hypoxic-ischemic brain injury in many small and large animal studies^36^. Allopurinol reduces oxidative stress and therefore early administration appears to be extremely important regarding its activity following neonatal hypoxic-ischemic brain injury^10^. Currently, a randomized-controlled clinical trial (ALBINO study, clinical trials gov: NCT03162653) is investigating the additional neuroprotective effects of allopurinol in combination with therapeutic hypothermia; pre-clinical data is still lacking to prove additional neuroprotection of allopurinol in combination with therapeutic hypothermia. We found that allopurinol at a concentration of 100 mg/kg significantly reduced brain area loss following pre-hypoxia administration, followed by four maintenance doses after HI every twelve hours (Table 1).

### Melatonin

Melatonin significantly reduces brain area loss following post-hypoxia treatment at a concentration of 25 mg/kg, followed by a single maintenance dose at the same concentration every 12 h for two consecutive days in our P7 model (Table 1). Melatonin (*N*-acetyl-5-methoxytryptamine), an indolamine hormone, is considered one of the most promising neuroprotective agents for NE, with preclinical studies confirming safety and efficacy as a single agent and as an adjunct with HT^37^. Melatonin’s diverse neuroprotective properties include antioxidant, anti-inflammatory, and anti-apoptotic effects^38,39^. Its strong safety profile, ability to cross the blood brain barrier and its diverse pleiotropic properties has led to a large number of preclinical animal studies supporting its neuroprotective efficacy (for review of neonatal studies see^37^). While the preclinical data are compelling, clinical studies in infants with NE are limited^38^. Over the past decade, the ability of melatonin to augment HT has been studied in the neonatal piglet model of perinatal asphyxia^40–43^. From this model, it is apparent that the neuroprotective effects of melatonin are time-critical and dose dependent. Therapeutic melatonin levels are likely to be 15–30 mg/L and for optimal effect, these need to be achieved within the first 6 h after birth.

As melatonin is sparingly soluble in aqueous vehicles, solubility enhancers are needed, such as ethanol or other excipients, to obtain a solution with the desired concentration. In our study, a melatonin formulation with sulfobutylether beta-cyclodextrin was used as an excipient. There are, however, safety concerns around the use of beta-cyclodextrin products in children and other formulations would need to be developed for clinical use^44^. Currently, there are melatonin/ethanol formulations under investigation which both achieve therapeutic levels of melatonin whilst limiting the blood alcohol concentration in line with FDA guidance on ethanol excipients for neonates (< 0.25 g/L).

### Clemastine

Clemastine is a widely used histamine H1 receptor antagonist which has been shown to be neuroprotective following experimental hypoxic brain injury in adult rodents^45^. The neuroprotective effects are mainly due to its anti-inflammatory properties in addition to an improvement of myelination and oligodendrocyte differentiation^46^. Clemastine has lately become a promising treatment option for patients suffering multiple sclerosis^47^. In an experimental neonatal study of chronic hypoxic brain injury, Clemastine was shown to rescue myelination defects and to promote functional recovery^48^. To date, Clemastine has never been used in neonatal hypoxic-ischemic brain injury studies; however, it has been demonstrated to play a key role in myelination and white matter regeneration after stroke^48,49^. Our study shows that Clemastine administration at a concentration of 10 mg/kg followed by a single daily maintenance dose at the same concentration for 6 consecutive days significantly reduces brain area loss following daily post-hypoxic treatment (Table 1).

### ß-Hydroxybutyrate

ß-Hydroxybutyrate (BHB) is a ketone body which might serve as alternative fuel to glucose following neonatal hypoxic ischemic brain injury^50^. During primary energy failure in the neonatal hypoxic-ischemic injured brain, energy demand is high and glucose supply is most likely insufficient. Therefore, the demand for alternative fuels is high. A benefit of BHB on improving cognitive performance as well as reduction of oxidative stress is linked to improved metabolic efficiency^51^. BHB has shown significant neuroprotection in an preclinical rat model of hypoxic-ischemic brain injury, reducing necrosis, improving brain injury and improving long-term functional outcome^52^. We found that BHB administration immediately after hypoxia at a concentration of 630 mg/kg and with a maintenance dose within the first 12 h post HI (Table 1), significantly improved brain area loss in our model, particularly when it is compared to the pooled HI/saline group. Of note, in the matched control experiment we found a non-significant probability of efficacy. However, compared to the pooled HI/saline control, we found a significant neuroprotective treatment effect with a probability of efficacy of more than 97%. As explained in the statistical methods, this difference can be explained by the individual variability of treatment efficacy in the original experiment compared to the widespread variability of the pooled HI/saline group^20,53,54^.

### Omegaven

Omegaven, an Omega-3 triglyceride emulsion, has been shown to be neuroprotective in different preclinical animal models of newborn brain injury^55^. Docosahexaenoic acid (DHA) is one of the major omega-3 polyunsaturated fatty acids and it has been shown to be essential for development and function of the brain. It reduces excitotoxicity, inflammation and free radical production following neonatal hypoxic-ischemic brain injury in different animal models^55^. We found that a single dose of Omegaven (0.75 mg/kg) (Table 1) administered immediately after hypoxia–ischemia was superior to a repetitive dosing regimen and significantly reduced brain area loss in our standardized model. We adopted the same treatment regimen as previously described^56,57^ to promote a neuroprotective effect. Using a comparable animal model, it was shown that DHA did not augment neuroprotection when combined with therapeutic hypothermia^58^. Omegaven is widely used in neonates and pediatric patients and has a safe drug profile in these patients^59,60^.

### Iodide

The experience using Iodide as a neuroprotectant following experimental brain injury is sparse. Iodide is an essential micronutrient. Being an integral component of thyroid hormone, it mediates the effects of thyroid hormone on brain development^61^. In addition, it has been shown to reduce inflammation and oxidative stress in experimental studies^61^. Therefore, it is of interest as neuroprotective treatment option following neonatal HI. We show here that Iodide at a concentration of 3 mg/kg administrated after ligation followed by two more maintenance doses every 24 h (Table 1) is neuroprotective following our well-established HI brain injury model, when compared against the pooled HI/saline group, with a probability of efficacy of 98.0%, whilst it was not considered neuroprotective when compared to the matched control group. Again, as explained in the statistical methods this difference can be explained by the individual variability of treatment efficacy in the original experiment compared to the widespread variability of the pooled HI/saline group^20,53,54^. Further experimental studies are needed to investigate the neuroprotective potency of Iodide.

### Other drug candidates

The goal of this study was to perform a head-to-head screen of compounds previously reported to have neuroprotective activity to identify candidates for more intensive future studies. Based on the published literature, all 25 putative therapeutic agents had shown promise as a therapy following experimental neonatal HI brain injury. Seventeen candidates failed to be neuroprotective in our study. This is most likely not due to the administration protocols, as we chose the best available treatment regiments based on the published literature. Reproducibility of research findings is crucial when aiming for translational treatment options. However, there are many examples showing failed reproducibility in research^14^. One aim to improve the robustness and reproducibility of research findings is experimental rigor and the report of experimental details, as presented in this study and often missing in the published literature. We have used the Vannucci model of unilateral HI brain injury in many studies and proven the therapeutic efficacy of HT in different studies, as shown by different labs in the last two decades^15^. Additionally, we used adequate group sizes for each individual treatment group, which should be sufficient to detect neuroprotective efficacy. Therefore, we believe that the experimental setup was not the reason for our failure to reproduce neuroprotection of the 17 candidates.

One of the candidates that failed to show neuroprotection in our study was erythropoietin (Epos). Indeed, neither erythropoietin nor darbepoietin significantly reduced brain injury in our study. Of notice, although Epo has shown benefit in rodent models in combination with HT, there was no augmentation of HT protection in the fetal sheep model^62^ or in the neonatal piglet^43^. In 2022, the Phase III Heal study did not show benefit of Epo as an adjunct to HT in 500 NE babies^13^. Whether Epo will remain to be a potential neuroprotective drug candidate in asphyxiated newborns in LMICs, remains unclear.

Another candidate that failed to show neuroprotection in our study is Azithromycin. Azithromycin, an antibiotic with wide use in neonates for many decades, has been reported to be neuroprotective following neonatal hypoxic-ischemic brain injury and inflammation-sensitized hypoxic–ischemic brain injury in newborn rats^63,64^. Several clinical trials demonstrated that Azithromycin crosses the placenta and accumulates in the fetus, reducing the risk of clinical infection and death^65–67^. Therefore, Azithromycin might be a potential neuroprotective treatment option, especially in LMIC, as it is safe, easy to deliver and cheap in cost. However, as we failed to prove efficacy in this study, additional experiments, including pharmacokinetics and dynamics using large animal models of hypoxic-ischemic brain injury are needed^68^, before it can be transferred to clinical studies in LMICs.

### Limitations

We aimed to administer treatment drug candidates based on the best available published evidence regarding time and dose of administration. The first treatment dose was administered before the initiation of hypoxia for drugs that were known to cross the placental barrier and could potentially be administered perinatally. In certain clinical scenarios this may not be feasible, especially in LMIC. However, our results reflect a primary screening of potential neuroprotective agents in the most optimal scenario (pre-hypoxia treatment). Subsequent studies may aim to investigate those pre-screened neuroprotective agents, to be administered in a more amenable clinical translation scenery. In addition, we did not investigate pharmacokinetics (PK) or -dynamics (PD) of the potential neuroprotective compounds in our model yet. These studies need to be performed before moving into large animal models and clinical trials optimizing the dosing regimen for each individual neuroprotective compound. Also, we did not test the drug candidates in combination with HT. As HT is not routinely available and failed to show neuroprotection in clinical studies 5 centres in India, Sri Lanka and Bangladesh^5^, we did not investigate the drug candidates in combination with HT. However, these future studies will need to be performed, before any promising treatment options (e.g., caffeine) can be offered to cooled asphyxiated newborns. Furthermore, we did not investigate long-term follow-up in the animals. This would have not been feasible for a study of this scale. Additionally, analysing brain area loss seven days after the insult might have failed to demonstrate neuroprotection of drug candidates that operate via alternative mechanisms of neuroregeneration. This might have been the case e.g., for cannabinoids, as they have been shown to be neuroprotective following long-term experimental outcome studies, even though early brain area loss is not reduced^69^. Lastly, we did not find a clear pattern of mechanism of action which might offer the best neuroprotection. Most of the used treatment drugs do have multiple mechanisms of action, such as reducing inflammation or oxidate stress. Due to the large number of treatment drugs used and because this was not the aim of this current study.

### Next steps

The overall aim of this large scale multi-drug randomized controlled animal screening trial was to compare different neuroprotective treatments using a standardized model. We have identified eight neuroprotective agents, of which seven showed superior neuroprotection compared to the currently available standard of care (HT). A priority in future research is to characterize the exact mechanisms of action of the most promising agents to identify potential combinatory treatment options. In addition, these eight candidates need to be investigated using large neonatal animal models (sheep and neonatal piglet) to demonstrate safety and efficacy before initiating their clinical use. We believe that these studies can be performed in a short time frame, leading to clinical studies within the next few years, especially in LMIC where HT appears to be not safe and the efficacy is uncertain.

### Summary

This first published large multi-drug randomized controlled preclinical screening trial confirms neuroprotection of eight therapeutic agents. The most promising candidates were caffeine and SAG, showing significantly high neuroprotection after HI. This screening trial provides sufficient evidence for progression to large animal models where safety, efficacy, pharmacokinetics and dynamics can be tested, before their use can be translated to clinical trials. Additionally, it is of high importance to further screen alternative treatments as melatonin which is being assessed in the inflammation sensitized HI models (rat and piglet), for its high relevance in LMIC as a readily accessible treatment.

## Supplementary Information

Supplementary Legends.

Supplementary Table 1.

Supplementary Table 2.

Supplementary Table 3.

## Data Availability

All data can be accessed via the corresponding author.
