# Supplementary material for: Comparing the efficacy in reducing brain injury of different neuroprotective agents following neonatal hypoxia–ischemia in newborn rats: a multi-drug randomized controlled screening trial
Source: Sci Rep. 2023 Jun 10;13:9467. doi: 10.1038/s41598-023-36653-9 (PMC10257179; doi:10.1038/s41598-023-36653-9)
Supplement: Supplementary file 1 — Supplementary Legends. [file 41598_2023_36653_MOESM1_ESM.docx]

**Supplementary Table 1:** Total list of 35 treatment compounds that were scored based on the evidence of efficacy.

**Supplementary Table 2:** List of used drug candidates with individual source identification.

**Supplementary Table 3:** Individual dosing and drug treatment intervals with corresponding literature references.
