# Supplementary material for: Comparing the efficacy in reducing brain injury of different neuroprotective agents following neonatal hypoxia–ischemia in newborn rats: a multi-drug randomized controlled screening trial
Source: Sci Rep. 2023 Jun 10;13:9467. doi: 10.1038/s41598-023-36653-9 (PMC10257179; doi:10.1038/s41598-023-36653-9)
Supplement: Supplementary file 2 — Supplementary Table 1. [file 41598_2023_36653_MOESM2_ESM.pdf]

	Primary Mechanism (1=ROS scavenger/ Antioxident / NOS; 2=Anti-Inflammatory / Anti-convulsant / Cellular neuron stabelizer; 3=Neuro-restoration / Stem cell stimulation / Perfusion; 4=Alternative fuel)	Additional Mechanism(s): (1=ROS scavenger/ Antioxident / NOS; 2=Anti-Inflammatory / Anti-convulsant / Cellular neuron stabelizer; 3=Neuro-restoration / Stem cell stimulation / Perfusion; 4=Alternative fuel)	Approved route of admin (1=oral; 2=intravenous; 3=ophthalmic)	Timing of intervention (1=antenatal; 2=intrapartum during delivery; 3=immediately postnatally; 4=delayed postnatally after diagnosis of NE)	Primary Score (1=best evidence for treatment efficacy; 9= no evidence for treatment efficacy)	Secondary Score	Average Score
**Melatonin**	1	2		2, 3	1,5	1,5	1,5
**N-acetylcysteine**	1	unknown		1, 2, 3, 4	3	2	2,5
**Sonic Hedgehog Agonist**	3	2		3	3	3	2,5
**Clemastine**	3	2	1	3,4	3	3	2,5
**Azithromycin**	2	3	1,2,3	2,3	2	4	3
**Carnitine**	4	1,2,3	1,2	3	3	3	3
**Caffeine**	3	unknown	1,2		3	3	3
**Magnesiumsulfate**	2	unknown		1,3	3	3	3
**Erythropoietin**	3	2	2		4	3	3,5
**Cannabinoids**	2	1,2	1	3,4	4	3	3,5
**Sildenafil**	3	unknown	1,2	4	3	4	3,5
**Darbepoetin**	3	2	2		3	5	4
**ISRIB**	2	unknown			2	6	4
**Uridine**	1	unknown	1	2,3	4	5	4,5
**Barbiturates**	2	unknown	1,2	4	6	3	4,5
**2-Iminobiotin**	1	unknown		3	5	5	5
**Iodide**	1	unknown	1,3	2	6	4	5
**Edaravone**	1	unknown	2	2,3	4	6	5
**Allopurinol**	1	unknown	1,2	2,3,4	4	6	5
**Dexmedetomidine**	2	1	1,2	3,4	4	6	5
**Topiramate**	2	unknown	1	4	5	6	5,5
**Tetrahydrobiopterin**	4	unknown		1	7	5	6
**Folic acid**	2	1	1	1	5,75	7	6,375
**B-hydroxy butyrate**	4	unknown			8	5	6,5
**Creatine**	4	unknown	1	1	6	7	6,5
**Mito-Q**	1	unknown		3	7	6	6,5
**Lactoferrin**	1	3			6,25	6,75	6,5
**Midkine**	2	1			7	6	6,5
**C-Jun N-terminal kinases**	2	1			7	7	7
**Osteopontin**	3	unknown			6,5	7,5	7
**Irisin**	2	unknown			7,5	7	7,25
**Thiorphan**	1	unknown	1		7,25	7,75	7,5
**LP17**	2	1			8,5	8	8,25
**Argon**	2	1			not applicable in LMIC	not applicable in LMIC	not applicable in LMIC
**Xenon**	2	1		3	not applicable in LMIC	not applicable in LMIC	not applicable in LMIC
