# Supplementary material for: Comparing the efficacy in reducing brain injury of different neuroprotective agents following neonatal hypoxia–ischemia in newborn rats: a multi-drug randomized controlled screening trial
Source: Sci Rep. 2023 Jun 10;13:9467. doi: 10.1038/s41598-023-36653-9 (PMC10257179; doi:10.1038/s41598-023-36653-9)
Supplement: Supplementary file 3 — Supplementary Table 2. [file 41598_2023_36653_MOESM3_ESM.pdf]

Drug	Company/Catnr.	Form	Stock solution 1 / Stock solution 2
2- Iminobiotin	Sigma 14632	Powder	NaCl 0.9%
Allopurinol	Cayman 1-8003649897	Powder	NaCl 0.9% / corn oil
Azithromycin	Sigma D6154	Powder	NaCl 0.9%
Beta-Hydroxylbutyrate	Sigma 298360	Powder	NaCl 0.9%
Caffeine	Cooper 03400934535932	Liquid	NaCl 0.9%
Canabidiol	Tocris 1570	Powder	NaCl 0.9%
Carnitine	Sigma A67066	Powder	NaCl 0.9%
Clemastine	Selleckchem S1847	Powder	NaCl 0.9%
Darbepoietin	Amgen 04150015282239	Liquid	NaCl 0.9%
Edaravone	Sigma M70800-59	Powder	NaCl 0.9%
Erythropoietin	Hexal 64150034381227	Liquid	NaCl 0.9%
Levetirazetam	Hikma 2105163.1	Liquid	NaCl 0.9%
Magnesiumsulfate	Inresa 4718mA	Powder	NaCl 0.9%
Melatonin	Chiesi C110358	Powder	NaCl 0.9%
Metformin	Sigma PHR1084	Powder	NaCl 0.9%
Mitoquinol	Cayman (800) 364-9897	Powder	NaCl 0.9%
N- Acetycystein	Sigma A7250	Powder	NaCl 0.9%
Omegaven	Fresenius C6453.0011	Liquid	Ready to use
Phenobarbital	Desitin 6245807.00.00	Liquid	NaCl 0.9%
Sildenafil	Pfizer 054115062332481	Liquid	Ready to use
Sodium Iodide	Sigma 409286	Powder	NaCl 0.9%
Sonic Hedgehog Agonist	Curis-201807	Powder	Kolliphor / NaCl 0.9%
Tetrahydrobipterin	Sigma T4425	Powder	NaCl 0.9%
Topiramate	Sigma 1672206	Powder	NaCl 0.9%
Uridine	Sigma U3750	Powder	NaCl 0.9%
