# Supplementary material for: Comparing the efficacy in reducing brain injury of different neuroprotective agents following neonatal hypoxia–ischemia in newborn rats: a multi-drug randomized controlled screening trial
Source: Sci Rep. 2023 Jun 10;13:9467. doi: 10.1038/s41598-023-36653-9 (PMC10257179; doi:10.1038/s41598-023-36653-9)
Supplement: Supplementary file 4 — Supplementary Table 3. [file 41598_2023_36653_MOESM4_ESM.pdf]

	Drug Dose Concentration Per Injection	Drug Dose Treatment	Reference
**2-Iminobiotin**	50 mg/kg	1h before hypoxia, 12, 24, 36, and 48 h after 1st dose	[1]
**Allopurinol**	100 mg/kg	1h before hypoxia, 12, 24, 36, and 48 h after 1st dose	[2]
**Azithromycin**	45 mg/kg 1st dose, then 22.5 mg/kg	1h before hypoxia, 24, and 48 h after 1st dose	[3, 4]
**Caffeine**	40 mg/kg	1h before hypoxia, 24, and 48 h after 1st dose	[5-8]
**Carnitine**	100 mg/kg	1h before hypoxia, 24, and 48 h after 1st dose	[9-11]
**Cannabidiol**	1 mg/kg	1h before hypoxia, 24, and 48 h after 1st dose	[12-14]
**Clemastine**	10 mg/kg	Immediately after Hypoxia-Ischemia, 24, 48, 72, 96, 120 and 144 h after 1st dose	[15, 16]
**Darbepoietin delayed treatment**	25 µg/kg	24 h after Hypoxia-Ischemia	[17]
**Darbepoietin immediate treatment**	25 µg/kg	1 h after Hypoxia-Ischemia	[17]
**Edaravone**	9 mg/kg	1h before hypoxia, 24, and 48 h after 1st dose	[18, 19]
**Erythropoietin delayed treatment**	5000 IE/kg	24, 48, and 72 h after Hypoxia-Ischemia	[20, 21]
**Erythropoietin immediate treatment**	5000 IE/kg	1 h after Hypoxia-Ischemia, 24, and 48 h after 1st dose	[20, 21]
**Iodide**	3 mg/kg	1h before hypoxia, 24, and 48 h after 1st dose	[22]
**Levetirazetam**	20 mg/kg	1h before hypoxia, 24, and 48 h after 1st dose	[23]
**Melatonin**	25 mg/kg	Immediately after Hypoxia-Ischemia, 12, 24, 36 and 48 h after 1st dose	[24-26]
**Metformin**	20 mg/kg	1h before hypoxia, 24, and 48 h after 1st dose	[27]
**Magnesiumsulfate**	500 mg/kg	1h before hypoxia, 24, and 48 h after 1st dose	[28-30]
**Mitoquinol**	0.4 mg/kg	1h before hypoxia, 12, 24, 36, and 48 h after 1st dose	[31, 32]
**N-Acetylcystein**	200 mg/kg	1h before hypoxia, 12, 24, 36, and 48 h after 1st dose	[33, 34]
**Omegaven**	750 mg/kg	Immediately after Hypoxia-Ischemia	[35, 36]
**Omegaven 2 doses**	750 mg/kg	Immediately after Hypoxia-Ischemia and 1 h after 1st dose	[35, 36]
**Phenobarbital**	20 mg/kg	1h before hypoxia, 24, and 48 h after 1st dose	[37]
**Sonic Hedgehog Agonist**	50 mg/kg	Immediately after Hypoxia-Ischemia	[38, 39]
**Sildenafil**	10 mg/kg	1h before hypoxia, 24, and 48 h after 1st dose	[40-42]
**ß-Hydroxybutyrate**	630 mg/kg	Immediately after Hypoxia-Ischemia, 2, 5 and 12 h after 1st dose	[43-45]
**Tetrahydrobiopterin**	20 mg/kg	Immediately after Hypoxia-Ischemia, 24 and 48 h after 1st dose	[46]
**Therapeutic Hypothermia**	-	Immediately after Hypoxia-Ischemia, body temperature 32°C for 5 h	[47, 48]
**Topiramate**	40 mg/kg	Immediately after Hypoxia-Ischemia, 12, 24, 36 and 48 h after 1st dose	[49-51]
**Uridine**	500 mg/kg	1h before hypoxia, 24 and 48 h after 1st dose	[52, 53]

1. Tweel, E.R.v.d., et al., *Long-Term Neuroprotection with 2-Iminobiotin, An Inhibitor of Neuronal and Inducible Nitric Oxide Synthase, after Cerebral Hypoxia-Ischemia in Neonatal Rats.* 2005. **25**(1): p. 67-74.
2. Durán Fernández-Feijóo, C., et al., *Effects of Hypothermia and Allopurinol on Oxidative Status in a Rat Model of Hypoxic Ischemic Encephalopathy.* 2021. **10**(10): p. 1523.
3. Barks, J.D.E., et al., *Azithromycin reduces inflammation-amplified hypoxic–ischemic brain injury in neonatal rats.* Pediatric Research, 2022. **92**(2): p. 415-423.
4. Barks, J.D.E., et al., *Repurposing azithromycin for neonatal neuroprotection.* Pediatric Research, 2019. **86**(4): p. 444-451.
5. Alexander, M., et al., *Therapeutic effect of caffeine treatment immediately following neonatal hypoxic-ischemic injury on spatial memory in male rats.* Brain Sci, 2013. **3**(1): p. 177-90.
6. Xu, F.L., et al., *[Effects of caffeine citrate on myelin basic protein in neonatal rats with hypoxic-ischemic brain damage].* Zhongguo Dang Dai Er Ke Za Zhi, 2015. **17**(9): p. 984-8.
7. Kilicdag, H., et al., *Effects of caffeine on neuronal apoptosis in neonatal hypoxic-ischemic brain injury.* The Journal of Maternal-Fetal & Neonatal Medicine, 2014. **27**(14): p. 1470-1475.
8. Soontarapornchai, K., et al., *Pharmacodynamic Effects of Standard versus High Caffeine Doses in the Developing Brain of Neonatal Rats Exposed to Intermittent Hypoxia.* 2021. **22**(7): p. 3473.
9. Wainwright, M.S., et al., *Carnitine treatment inhibits increases in cerebral carnitine esters and glutamate detected by mass spectrometry after hypoxia-ischemia in newborn rats.* Stroke, 2006. **37**(2): p. 524-30.
10. Wainwright, M.S., et al., *L-Carnitine Reduces Brain Injury after Hypoxia-Ischemia in Newborn Rats.* Pediatric Research, 2003. **54**(5): p. 688-695.
11. Demarest, T.G., et al., *Sex dependent alterations in mitochondrial electron transport chain proteins following neonatal rat cerebral hypoxic-ischemia.* Journal of Bioenergetics and Biomembranes, 2016. **48**(6): p. 591-598.
12. Pazos, M.R., et al., *Cannabidiol administration after hypoxia–ischemia to newborn rats reduces long-term brain injury and restores neurobehavioral function.* Neuropharmacology, 2012. **63**(5): p. 776-783.
13. Ceprián, M., et al., *Cannabidiol Administration Prevents Hypoxia-Ischemia-Induced Hypomyelination in Newborn Rats.* Front Pharmacol, 2019. **10**: p. 1131.
14. Ceprián Costoso, M., *Caracterización de los efectos neuroprotectores del cannabidiol en el daño cerebral hipóxico-isquémico neonatal.* 2019.
15. Cree, B.A.C., et al., *Clemastine rescues myelination defects and promotes functional recovery in hypoxic brain injury.* Brain, 2018. **141**(1): p. 85-98.
16. Xie, D., et al., *Clemastine improves hypomyelination in rats with hypoxic–ischemic brain injury by reducing microglia-derived IL-1β via P38 signaling pathway.* Journal of Neuroinflammation, 2020. **17**(1).
17. Belayev, L., et al., *Neuroprotective Effect of Darbepoetin Alfa, a Novel Recombinant Erythropoietic Protein, in Focal Cerebral Ischemia in Rats.* 2005. **36**(5): p. 1065-1070.

18. Takizawa, Y., et al., *Edaravone Inhibits DNA Peroxidation and Neuronal Cell Death in Neonatal Hypoxic-Ischemic Encephalopathy Model Rat.* Pediatric Research, 2009. **65**(6): p. 636-641.
19. Zhou, S., et al., *Neuroprotective effects of edaravone on cognitive deficit, oxidative stress and tau hyperphosphorylation induced by intracerebroventricular streptozotocin in rats.* NeuroToxicology, 2013. **38**: p. 136-145.
20. Fan, X., et al., *Hypothermia and erythropoietin for neuroprotection after neonatal brain damage.* Pediatric Research, 2013. **73**(1): p. 18-23.
21. Fang, A.Y., et al., *Effects of combination therapy using hypothermia and erythropoietin in a rat model of neonatal hypoxia–ischemia.* Pediatric Research, 2013. **73**(1): p. 12-17.
22. Brandt, M.J.V., et al., *Nutritional Supplementation Reduces Lesion Size and Neuroinflammation in a Sex-Dependent Manner in a Mouse Model of Perinatal Hypoxic-Ischemic Brain Injury.* Nutrients, 2021. **14**(1).
23. Komur, M., et al., *Neuroprotective effect of levetiracetam on hypoxic ischemic brain injury in neonatal rats.* Childs Nerv Syst, 2014. **30**(6): p. 1001-9.
24. Pang, R., et al., *Melatonin and/or erythropoietin combined with hypothermia in a piglet model of perinatal asphyxia.* Brain Commun, 2021. **3**(1): p. fcaa211.
25. Berger, H.R., et al., *Early metabolite changes after melatonin treatment in neonatal rats with hypoxic-ischemic brain injury studied by in-vivo1H MR spectroscopy.* PLoS One, 2017. **12**(9): p. e0185202.
26. Xu, L.-X., et al., *Melatonin alleviates brain and peripheral tissue edema in a neonatal rat model of hypoxic-ischemic brain damage: the involvement of edema related proteins.* BMC Pediatrics, 2017. **17**(1): p. 90.
27. Skemiene, K., et al., *Comparison of Effects of Metformin, Phenformin, and Inhibitors of Mitochondrial Complex I on Mitochondrial Permeability Transition and Ischemic Brain Injury.* 2020. **10**(10): p. 1400.
28. Cetinkaya, M., et al., *Possible neuroprotective effects of magnesium sulfate and melatonin as both pre- and post-treatment in a neonatal hypoxic-ischemic rat model.* Neonatology, 2011. **99**(4): p. 302-10.
29. Galvin, K.A. and D.E. Oorschot, *Postinjury Magnesium Sulfate Treatment Is Not Markedly Neuroprotective for Striatal Medium Spiny Neurons after Perinatal Hypoxia/Ischemia in the Rat.* Pediatric Research, 1998. **44**(5): p. 740-745.
30. Seyama, T., et al., *Pretreatment with magnesium sulfate attenuates white matter damage by preventing cell death of developing oligodendrocytes.* J Obstet Gynaecol Res, 2018. **44**(4): p. 601-607.
31. Hobbs, C.E., et al., *Neonatal rat hypoxia-ischemia: Effect of the anti-oxidant mitoquinol, and S-PBN.* Pediatr Int, 2008. **50**(4): p. 481-8.
32. Nuzzo, A.M., et al., *Placental Adaptation to Early-Onset Hypoxic Pregnancy and Mitochondria-Targeted Antioxidant Therapy in a Rodent Model.* The American Journal of Pathology, 2018. **188**(12): p. 2704-2716.
33. Nance, E., et al., *Systemic dendrimer-drug treatment of ischemia-induced neonatal white matter injury.* J Control Release, 2015. **214**: p. 112-20.
34. Nemeth, C.L., et al., *Uptake of dendrimer-drug by different cell types in the hippocampus after hypoxic-ischemic insult in neonatal mice: Effects of injury, microglial activation and hypothermia.* Nanomedicine, 2017. **13**(7): p. 2359-2369.

35. Williams, J.J., et al., *N-3 fatty acid rich triglyceride emulsions are neuroprotective after cerebral hypoxic-ischemic injury in neonatal mice.* PLoS One, 2013. **8**(2): p. e56233.
36. Manual Kollareth, D.J., et al., *Acute injection of a DHA triglyceride emulsion after hypoxic-ischemic brain injury in mice increases both DHA and EPA levels in blood and brain☆.* Prostaglandins, Leukotrienes and Essential Fatty Acids, 2020. **162**.
37. Barks, J.D., et al., *Phenobarbital Augments Hypothermic Neuroprotection.* Pediatric Research, 2010. **67**(5): p. 532-537.
38. Nguyen, V., et al., *Neuroprotective effects of Sonic hedgehog agonist SAG in a rat model of neonatal stroke.* Pediatric Research, 2021. **90**(6): p. 1161-1170.
39. Yin, S., et al., *Neuroprotective Effects of the Sonic Hedgehog Signaling Pathway in Ischemic Injury through Promotion of Synaptic and Neuronal Health.* Neural Plasticity, 2020. **2020**: p. 8815195.
40. Yazdani, A., et al., *Sildenafil Improves Brain Injury Recovery following Term Neonatal Hypoxia-Ischemia in Male Rat Pups.* Developmental Neuroscience, 2016. **38**(4): p. 251-263.
41. Rogido, M., et al., *552 Does Sildenafil Protects the Developing Brain From Hypoxia- Induced Cell Death?* Pediatric Research, 2010. **68**(1): p. 283-283.
42. Léger, P., et al., *ABSTRACT 9: SILDENAFIL: ®: REDUCES DAMAGE OF HYPOXIA-ISCHEMIA AND IMPROVES LOCOMOTION BY THE RECRUITMENT OF COLLATERAL.* Pediatric Critical Care Medicine, 2014. **15**(4_suppl).
43. Lee, B.S., et al., *Exogenous β-Hydroxybutyrate Treatment and Neuroprotection in a Suckling Rat Model of Hypoxic-Ischemic Encephalopathy.* Developmental Neuroscience, 2018. **40**(1): p. 73-83.
44. Odorcyk, F.K., et al., *Differential glucose and beta-hydroxybutyrate metabolism confers an intrinsic neuroprotection to the immature brain in a rat model of neonatal hypoxia ischemia.* Experimental Neurology, 2020. **330**: p. 113317.
45. Puchowicz, M.A., et al., *Neuroprotection in diet-induced ketotic rat brain after focal ischemia.* J Cereb Blood Flow Metab, 2008. **28**(12): p. 1907-16.
46. Fabian, R.H., J.R. Perez-Polo, and T.A. Kent, *Perivascular nitric oxide and superoxide in neonatal cerebral hypoxia-ischemia.* Am J Physiol Heart Circ Physiol, 2008. **295**(4): p. H1809-14.
47. Wood, T., et al., *Treatment temperature and insult severity influence the neuroprotective effects of therapeutic hypothermia.* Sci Rep, 2016. **6**: p. 23430.
48. Sabir, H., et al., *Immediate hypothermia is not neuroprotective after severe hypoxia-ischemia and is deleterious when delayed by 12 hours in neonatal rats.* Stroke, 2012. **43**(12): p. 3364-70.
49. Clark, A.M., et al., *Plasma topiramate concentrations resulting from doses associated with neuroprotection against white matter injury and stroke in two strains of rat pups.* Pediatric Research, 2013. **73**(3): p. 317-324.
50. Liu, C., et al., *Neuroprotective effect of memantine combined with topiramate in hypoxic–ischemic brain injury.* Brain Research, 2009. **1282**: p. 173-182.
51. Liu, Y., et al., *Topiramate Extends the Therapeutic Window for Hypothermia-Mediated Neuroprotection After Stroke in Neonatal Rats.* 2004. **35**(6): p. 1460-1465.

52. Koyuncuoglu, T., et al., *Uridine protects against hypoxic-ischemic brain injury by reducing histone deacetylase activity in neonatal rats.* Restorative Neurology and Neuroscience, 2015. **33**: p. 777-784.
53. Goren, B., et al., *Long-term cognitive effects of uridine treatment in a neonatal rat model of hypoxic-ischemic encephalopathy.* Brain Research, 2017. **1659**: p. 81-87.
